# Restricted Genetic Variation in Populations of *Achatina* (*Lissachatina*) *fulica* outside of East Africa and the Indian Ocean Islands Points to the Indian Ocean Islands as the Earliest Known Common Source

**DOI:** 10.1371/journal.pone.0105151

**Published:** 2014-09-09

**Authors:** Ian Kendrich C. Fontanilla, Inna Mikaella P. Sta. Maria, James Rainier M. Garcia, Hemant Ghate, Fred Naggs, Christopher M. Wade

**Affiliations:** 1 School of Biology, University of Nottingham, University Park, Nottingham, United Kingdom; 2 DNA Barcoding Laboratory, Institute of Biology, University of the Philippines, Diliman, Quezon City, Philippines; 3 Department of Zoology, Modern College, Shivajinagar, Pune, India; 4 Department of Zoology, The Natural History Museum, London, United Kingdom; Australian Museum, Australia

## Abstract

The Giant African Land Snail, *Achatina* ( = *Lissachatina*) *fulica* Bowdich, 1822, is a tropical crop pest species with a widespread distribution across East Africa, the Indian subcontinent, Southeast Asia, the Pacific, the Caribbean, and North and South America. Its current distribution is attributed primarily to the introduction of the snail to new areas by Man within the last 200 years. This study determined the extent of genetic diversity in global *A. fulica* populations using the mitochondrial 16S ribosomal RNA gene. A total of 560 individuals were evaluated from 39 global populations obtained from 26 territories. Results reveal 18 distinct *A. fulica* haplotypes; 14 are found in East Africa and the Indian Ocean islands, but only two haplotypes from the Indian Ocean islands emerged from this region, the C haplotype, now distributed across the tropics, and the D haplotype in Ecuador and Bolivia. Haplotype E from the Philippines, F from New Caledonia and Barbados, O from India and Q from Ecuador are variants of the emergent C haplotype. For the non-native populations, the lack of genetic variation points to founder effects due to the lack of multiple introductions from the native range. Our current data could only point with certainty to the Indian Ocean islands as the earliest known common source of *A. fulica* across the globe, which necessitates further sampling in East Africa to determine the source populations of the emergent haplotypes.

## Introduction

Within the last 200 years, the Giant African Land Snail *Achatina* ( = *Lissachatina*) *fulica* Bowdich, 1822 has spread rapidly across the tropics from its native range in East Africa. It is now found across Africa, the Indian subcontinent, Southeast Asia, the Pacific, the Caribbean, and North and South America. *Achatina fulica* is a serious crop pest causing significant damage to vegetables and other food crops [1], [2] and serves as the intermediate host of the rat lungworm *Angiostrongylus cantonensis* that causes eosinophilic meningitis in humans [3]; the spread of the parasite is also affected by the dispersal of its snail host, as seen in the case of *A. fulica* in the Pacific [4]. The International Union for Conservation of Nature (IUCN) has listed *A. fulica* in its 100 most invasive species [5], and among the land snails, it is probably the most invasive [1]. Its status as a major agricultural pest is probably attributable to its high reproductive capacity and its generalist food behavior, feeding on a wide range of plants and detritus [1]. The tendency of people to transport the snails and release them into the wild either intentionally or inadvertently provides available pathways of dispersal that helps to spread them further [6], [7].

*Achatina fulica* is indigenous to the coast of East Africa but was introduced into the nearby Indian Ocean islands of Madagascar, Mauritius, the Comoros, Mayotte and Reunion prior to 1800 [8]. The snail is believed to have been brought to India in 1847 and to Sri Lanka in the early 1900's by naturalists [3], [9] and subsequently spread to Nepal to the north [10] and, via sea routes in cargo vessels, to the Malay Peninsula to the southeast [11]. Immediately before and during the Second World War, Japanese merchants and soldiers spread *A. fulica* further into Southeast Asia, Taiwan, and many islands of the Pacific [12]. By the late 1980's, *A. fulica* had been reported in the Caribbean, particularly in Guadeloupe and Martinique [13], in St. Lucia and Barbados by 2000 [14] and in Antigua by 2008 [15]. By the 1990's, *A. fulica* had already established itself in South America, particularly Brazil [7], [16], [17], Colombia, Ecuador, Peru [18], and most recently in Argentina [19]. The snail has also been observed in West and Northwest Africa in the Ivory Coast, Ghana and Morocco [1]. Although *A. fulica* has reached Australia, Japan, and the United States mainland in the past, authorities in these countries have successfully eradicated *A. fulica* for fear of its potential for causing damage to agriculture [11], [12]. In the United States alone, it took ten years and $1 million USD to completely eradicate the snail in Florida after its first introduction in 1966 [20]. However, the Florida Department of Agriculture and Consumer Services (FDACS) reported the resurgence of the snail in 2011 in the Miami-Dade area of Florida, USA due to illegal introductions, with 128,000 individuals eliminated in a span of two years. As an exotic pet [12], *A. fulica* is also present in temperate countries such as the UK and France.

Most exotic or invading species undergo founding events by a few individuals with low genetic variation; this could lead over time to a population bottleneck, causing massive reduction in genetic variation [21]. This was seen in the freshwater snail *Potamopyrgus antipodorum* introduced in Europe based on the 16S rRNA gene [22] and the apple snails *Pila conica* and *Pomacea canaliculata* introduced in Hawaii based on the COI and ND6 genes [23]. However, loss of genetic variation could be offset if multiple introductions from various native sources occur [24]. For example, high genetic diversity in the COI gene was observed for *Pomacea canaliculata* introduced in Asia, perhaps as a result of multiple introductions from its native range in Argentina [25].

Despite the detailed historical and anecdotal information concerning the spread of *Achatina fulica*, no systematic evaluation of its genetic variation across global populations has been undertaken. The extent of genetic variation within introduced populations remains unknown, and it is not known whether multiple introductions of *A. fulica* from different sources have occurred in any part of its new range. Using the mitochondrial 16S rRNA gene as a marker, this study aimed to assess genetic variation in global populations of *Achatina fulica*. A comparison of recently dispersed *A. fulica* populations from across the globe with those from East Africa and adjacent islands was undertaken, with populations examined from East Africa, the Indian Ocean islands, the Indian subcontinent, Southeast Asia, the Pacific, the Caribbean, and the Americas.

## Materials and Methods

### Sample collection

In total, 560 individuals of *Achatina fulica* from 39 populations in 26 territories across the globe were sampled (see Table 1). Efforts were made to ensure that major areas where the snails have been reported were sampled, i.e. East Africa (3 populations), Indian Ocean islands (4), Indian subcontinent (6), Southeast Asia (9), the Pacific islands (5), the Caribbean (2), North America (1) and South America (9). All samples were collected in public lands, and no permits were required in all countries covered in the study during sampling as these snails were not considered as endangered.

**Table 1:** Locality, collector and sample size of global populations of *Achatina fulica* and the summary of the distribution and frequency of the 16S rRNA haplotypes.

Locality	Collector	Sample size (no. of haplotypes)	Haplotype name (no. of individuals)
EAST AFRICA			
1) Kampala, Uganda	B. Rowson	5 (1)	O (5)
2) Dar Es Salaam, Tanzania	C. Ngereza	38 (6)	I (11), J (1), K (12),
			L (10), M (2), N (2)
3) Unknown, Tanzania	Anon.	2 (2)	J (1), R (1)
INDIAN OCEAN ISLANDS			
4) Mayotte	F. Barthelat	50 (6)	A (1), B (1), C (6),
			D (40), G (1), H (1)
5) Souillac, Mauritius	O. Griffiths	45 (3)	C (34), D (9), H (2)
6) Mahe, Seychelles	J. Gerlach	2 (1)	C (2)
7) Miaranony, East of Ranomafana National Park, Madagascar	K. Emberton	2 (1)	C (2)
INDIAN SUBCONTINENT			
8) Bharatpur, Nepal	P. Budha	22 (1)	C (22)
9) Nagpur, Maharashtra State, India	H. Ghate	7 (1)	P (7)
10) Nashik, Maharashtra State, India	H. Ghate	1 (1)	P (1)
11) Pune, Maharahtra State, India	H. Ghate	3 (1)	C (3)
12) Talegaon, Maharashtra State, India	H. Ghate	1 (1)	C (1)
13) Sri Lanka	Anon.	20 (1)	C (20)
SOUTHEAST ASIA			
14) Yangon, Myanmar/Burma	F. Naggs	20 (1)	C (20)
15) TrokNong Area, Chantaburi, Thailand	S. Panha & C. Sutcharit	20 (1)	C (20)
16) Phuket, Thailand	C. Wade & B. Rowson	20 (1)	C (20)
17) Quezon City, Philippines	I. Fontanilla	20 (1)	C (20)
18) Los Baños, Philippines	M. Carandang	20 (2)	C (17), E (3)
19) Singapore	M. Posa	20 (1)	C (20)
20) Penang, Malaysia	C. Wade	19 (1)	C (19)
21) Kota Kinabalu, Sabah, Borneo, Malaysia	M. Schilthuizen & T. Liew	20 (1)	C (20)
22) Cuc Phong National Park, Vietnam	C. Wade	4 (1)	C (4)
PACIFIC ISLANDS			
23) Hahasima, Ogasawara/Bonin	A. Davison	12 (1)	C (12)
24) Noumea, New Caledonia	C. Wade	21 (1)	F (21)
25) Moaroa Valley,Tahiti, French Polynesia	T. Coote	5 (1)	C (5)
26) Haapiti Valley, Moorea, French Polynesia	T. Coote	10 (1)	C (10)
27) Kaneohe, Oahu, Hawaii	K. Hayes	20 (1)	C (20)
CARIBBEAN			
28) Martinique	F. Adnai	20 (1)	C (20)
(captive bred F1 population, Nancy, Lorraine, France)			
29) Barbados	A. Norville	12 (1)	F (12)
NORTH AMERICA			
30) Florida, USA	F.J. Zimmerman	75 (1)	C (75)
SOUTH AMERICA			
31) Guayas, Ecuador	AgroCalidad Personnel	5 (2)	C (4), D (1)
32) La Troncar, Cañal, Ecuador	AgroCalidad Personnel	2 (1)	C (2)
33) Santo Domingo de los Tsáchiles, Ecuador	AgroCalidad Personnel	2 (1)	C (2)
34) Machala, El Oro, Ecuador	AgroCalidad Personnel	2 (1)	C (2)
35) La Mana, Cotopaxi, Ecuador	AgroCalidad Personnel	5 (2)	C (1), D (4)
36) Guaranda, Bolivar, Ecuador	AgroCalidad Personnel	2 (1)	C (2)
37) La Libertad, Santa Elena, Ecuador	AgroCalidad Personnel	1 (1)	Q (1)
38) Puerto Suarez, Bolivia	SENASAG Personnel	2 (2)	C (1), D (1)
39) Foz do Iguaçu, Paraná, Brazil	D.G. Robinson	3 (1)	C (3)
**TOTAL**		**560 (18)**	

### DNA extraction

Tissue from the foot muscle (approximately 8 mm^3^) was cut from each snail and subjected to DNA extraction. The first stage employed the NaOH direct lysis of the tissue based on the combined protocols of Jeanpierre [26] and Wang et al. [27] followed by a second stage involving the DNA purification and concentration method of Moore [28]; both stages were modified as follows. Tissue slices were ground in microfuge tubes with 400 µl of 0.1 N NaOH using sterile glass beads and a plastic pestle. The samples were heated at 95–100°C for 20 min then 200 µl of sterile distilled water and 600 µl of chloroform-isoamyl alcohol (24:1) were added. The tubes were inverted several times then centrifuged at 16,276 x g for 10 minutes. The upper phase (∼600 µl) was transferred into new tubes and an equal volume of isopropanol (∼600 µl) was added. The tubes were inverted several times then stored at −80°C for at least one hour. The tubes were centrifuged at 16,276 x g for 15 min, after which the isopropanol was carefully removed. The pellets were washed with 1 ml of 70% ice-cold ethanol and then centrifuged for 5 min at 16,276 x g. The ethanol was carefully removed and the DNA pellets were air-dried on a heat block at 45°C, after which they were re-suspended in 300 µl TE buffer (10 mMTris-HCl, 1 mM EDTA, pH 8.5).

### PCR and SSCP analysis using the 16S rRNA gene

For the 16S rRNA gene, primers 16S1i (5′-TGACTGTGCAAAGGTAGCATAA-3′) and 16S_SSCP2i (5′-CCTAGTCCAACATCGAGGTC-3′) were used to amplify a 293 bp fragment. This corresponds to domain IV of the 16S rRNA secondary structure [29]; a region previously employed by Stadler et al. [22] and Pinceel et al. [30] to assess genetic variation in populations of the aquatic snail *Potamopyrgus antipodorum* and the slug *Arion subfucus*, respectively. The PCR amplification was carried with 0.2 µM each of the primer, 1.5 mM MgCl_2_, 0.5 U Taq and 2 µl DNA sample in a final 50 µl volume. The PCR conditions were: 94°C for 2 min and 38 cycles of 94°C for 30 s, 45°C for 30 s and 65°C for 1 min.

The amplified PCR products were then subjected to SSCP analysis [31] using a native polyacrylamide gel system (7.5 ml 2X polyacrylamide gel solution, USA, 18 ml 1X TBE buffer, 4 ml distilled water, 40 µl TEMED, and 400 µl ammonium persulfate) at 180 V for 24 hours at 4°C. The bands were visualized using silver staining, after which haplotypes were identified and scored. Multiple individuals bearing unique haplotype gel profiles were checked for nucleotide differences by re-amplifying the PCR product from the DNA sample and sequencing. Sequencing was undertaken at Oxford Sequencing Service Facility, UK or 1^st^ Base, Singapore and the sequences were subsequently assembled in the STADEN package version 1.5.3 [32] and aligned manually within GDE Version 2.2 [33].

Each distinct sequence was identified as a haplotype and its frequency was scored. The haplotype and nucleotide diversities were then computed for the global data and for specific regions using DNAsp version 5 [34].

### Network analysis of the 16S rRNA haplotypes

To determine the evolutionary relationships of the different 16S haplotypes, a median joining network [35] of the haplotypes was also drawn using the Network version 4.502 program (http://www.fluxus-engineering.com).

## Results

**Figure 1:**
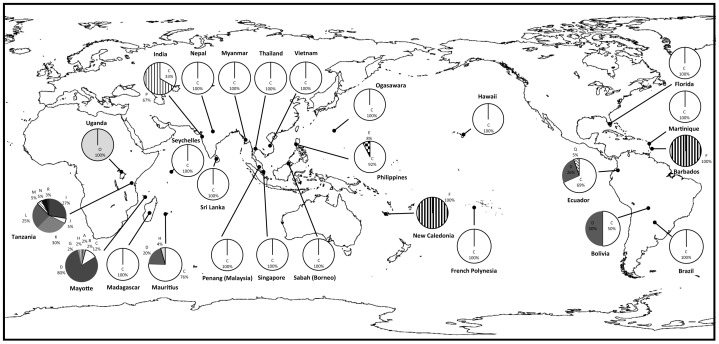
Distribution map of the *Achatina fulica* populations and their 16S rRNA haplotypes.

Eighteen distinct haplotypes (A-R) were detected (Table 1). To verify the accuracy of SSCP results, a total of 253 individuals representing these haplotypes were sequenced; in all cases, the identified SSCP haplotypes conformed to the DNA sequence, and there were no instances where the SSCP misidentified or missed any haplotypes. The 18 haplotypes varied at 20 sites (Table 2). Eight haplotypes were found in the two East African countries: Uganda with one haplotype (O) and Tanzania with seven haplotypes (I, J, K, L, M, N, and R, with K being the most numerous at 30%); mean haplotype diversity for the East African populations based on these two countries was 0.797 with a mean nucleotide diversity of 0.012. Among the Indian Ocean islands, Mayotte yielded the highest number of haplotypes with six (A, B, C, D, G and H, with D having the highest frequency 80%); three of these haplotypes were also found in Mauritius (C, D and H, with C having the highest frequency 76%) and only one was found in Madagascar and the Seychelles (C). Mean haplotype diversity for the Indian Ocean island populations was 0.535 whereas nucleotide diversity was 0.002. Of the haplotypes found in Africa and the Indian Ocean islands, only haplotypes C and D were found in populations outside of these areas; the majority carried haplotype C (88% frequency), while both haplotypes C and D were found in Bolivia and Ecuador in South America. Four unique non-African/Indian Ocean haplotypes were also detected: haplotype E in Los Baños, Philippines (15%) where it was found alongside haplotype C; haplotype F in New Caledonia in the Pacific (100%) and Barbados in the Caribbean (100%); haplotype P in India (67%) where it was found alongside haplotype C; and haplotype Q in Ecuador (5%) where it was found alongside haplotypes C and D. Mean haplotype diversity for areas outside East Africa and the Indian Ocean islands was 0.205 while nucleotide diversity was 0.001. Overall haplotype diversity for all populations was 0.445 whereas nucleotide diversity was 0.003. The frequencies and distribution of haplotypes are summarized in Table 1 and Figure 1.

**Table 2:** Twenty variable sites across the 18 haplotypes based on the 293-bp 16S rRNA fragment.

Haplotype		Nucleotide Position																			*N*
	19	42	71	102	103	106	145	151	155	156	158	197	205	210	217	258	281	282	285	286	
***A***	G	A	T	C	C	C	C	A	T	A	A	T	T	C	A	A	T	T	T	T	1
***B***	A	G	.	.	.	.	.	.	.	.	.	.	.	.	.	.	.	.	.	.	1
***C***	A	.	.	.	.	.	.	.	.	.	G	.	.	.	.	.	.	.	.	-	409
***D***	A	.	.	.	.	.	.	.	.	.	.	.	.	.	.	.	.	.	.	-	55
***E***	A	C	.	.	.	.	.	.	.	.	G	.	.	.	.	.	.	.	.	-	3
***F***	A	.	.	.	.	.	.	.	C	.	G	.	.	.	.	.	.	.	.	-	33
***G***	A	.	.	.	.	T	.	.	.	.	.	.	.	.	.	.	.	.	.	-	1
***H***	A	.	.	.	.	.	.	.	.	.	G	.	.	.	.	.	.	.	.	.	3
***I***	A	.	.	T	.	.	.	G	.	T	.	C	.	.	.	.	.	.	.	.	11
***J***	A	.	.	T	.	.	.	G	.	T	.	.	.	.	.	.	.	A	.	-	2
***K***	A	.	.	.	.	.	.	.	.	.	.	.	.	.	.	G	.	.	-	-	12
***L***	A	.	.	G	.	.	.	G	.	T	.	C	C	.	.	.	.	.	-	-	10
***M***	A	.	.	T	.	.	.	G	.	T	.	C	.	.	.	.	.	.	.	-	2
***N***	A	.	.	T	.	.	.	G	.	T	.	.	.	.	G	.	.	.	.	-	2
***O***	A	.	.	.	.	.	T	.	.	.	.	.	.	.	.	.	A	.	.	-	5
***P***	A	.	.	.	T	.	.	.	.	.	G	.	.	.	.	.	.	.	.	-	8
***Q***	A	.	C	.	.	.	.	.	.	.	G	.	.	.	.	.	.	.	.	-	1
***R***	A	.	.	.	.	.	.	.	.	.	.	.	.	A	.	.	.	.	-	-	1

A dot represents an identical nucleotide with respect to the reference haplotype *A*. *N* represents the number of individuals sampled for each haplotype.

**Figure 2:**
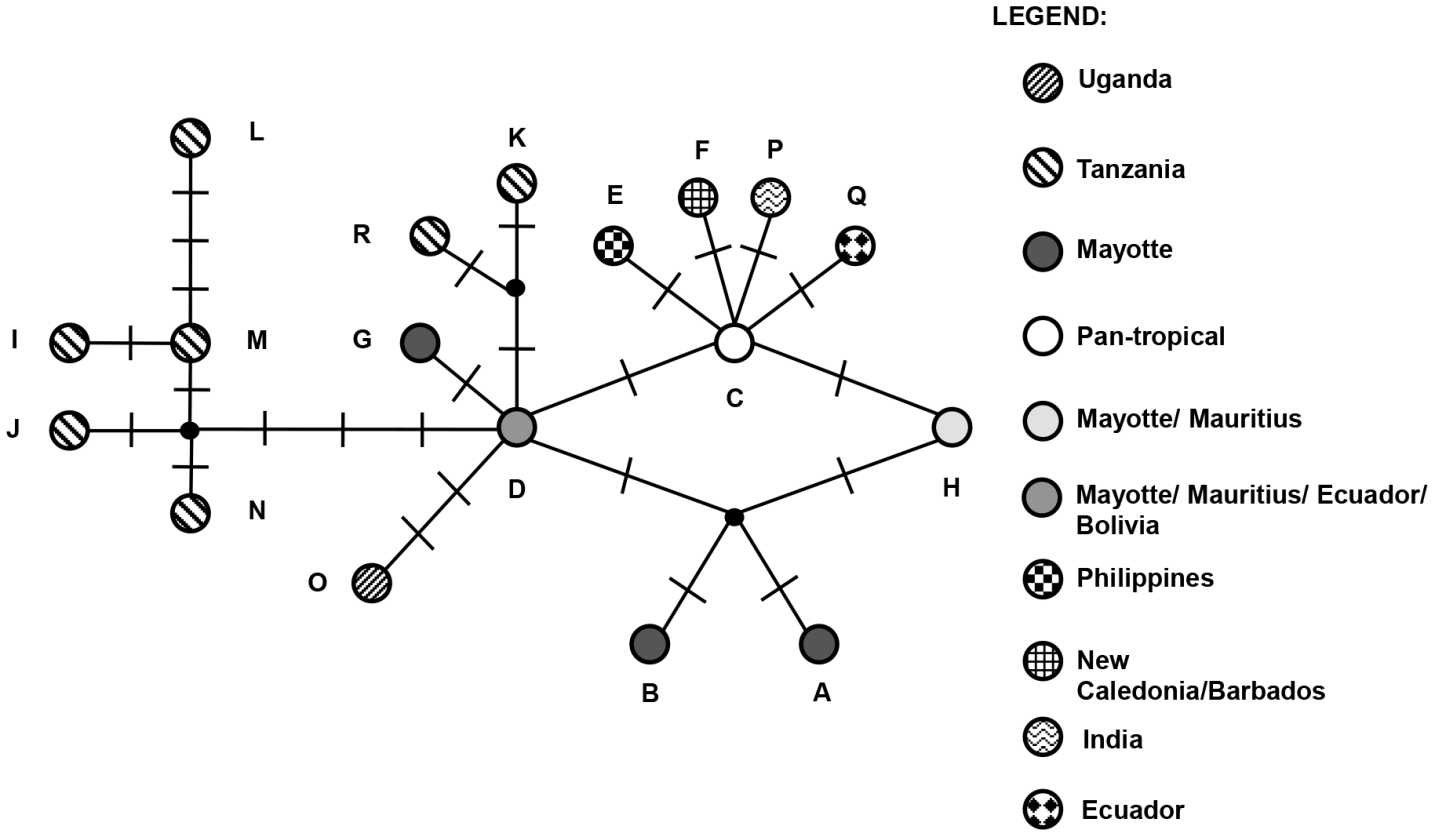
Median-joining network of the 18 *Achatina fulica* 16S haplotypes. Each mutation between haplotypes is represented by a bar. Unsampled putative haplotypes are represented by black dots.

Median network analysis (Figure 2) showed that five out of seven Tanzanian haplotypes (I, J, L, M and N) grouped together in the network analysis where they were linked to the Mayotte haplotype D with three mutations. The Tanzanian haplotypes K and R were also linked to haplotype D by two mutational steps each but were separated from the other Tanzanian haplotypes. Other haplotypes linked to the Mayotte/Mauritius/Ecuador haplotype D were the Ugandan haplotype O with two mutational steps, Mayotte haplotype G and the pantropical haplotype C with one mutational step each. Mayotte haplotype H, in turn, was linked to haplotype C by one mutation. Mayotte haplotypes A and B probably originated independently by one substitutional step from a hypothetical haplotype, which, in turn, was probably only one mutational step away from either D or H. The non-African/Indian Ocean haplotypes (E, F, P and Q) arose through a single mutation each from the pantropical haplotype C.

## Discussion

The distribution map of the *A. fulica* 16S haplotypes indicated that the majority (14) of the haplotypes are found in East Africa and the Indian Ocean islands and that only two of the sampled Indian Ocean haplotypes emerged from these areas, predominantly the C haplotype globally and the D haplotype in Ecuador and Bolivia. Four other haplotypes (E, F, P and Q) were not sampled in East Africa or the Indian Ocean islands and, with the exception of F, were represented by only a few individuals. *Achatina fulica* clearly exhibits little variation outside its native East Africa and the neighbouring Indian Ocean islands. The low variation outside of these areas could be attributed to founder effects where a population arose from a few introduced individuals into a new area; this is supported by low haplotype and nucleotide diversities, both with values less than 0.5 [36].

In India, historical records suggest that the prevalence of *Achatina fulica* could be traced back to the introduction of two individuals from Mauritius to Calcutta by the malacologist William Benson in 1847 [9]. Benson's friend released these snails into his garden [9], which then began to proliferate and slowly spread to the rest of India [37], [38] and later to Nepal [1], [10]. Although samples were not available from Calcutta, Indian populations from Maharashtra state harbour haplotype C and its related haplotype P. Haplotype C is also present in Nepal. Haplotype C is the most common haplotype in Mauritius, the source of Benson's *A. fulica*. The possibility of other introduction events in India should not be ruled out, particularly if we consider the trading history of India with many other countries in the past. In Sri Lanka, only haplotype C was observed, although data were obtained from only one population. The presence of the pantropical haplotype in Sri Lanka is not surprising since the Giant African Land Snail was introduced separately in 1900 by Oliver Collet, who sourced his snails either from India or Mauritius [11], which also have the C haplotype. The *A. fulica* populations from Peninsular Malaysia were known to be derived from Sri Lanka [11]; Peninsular Malaysia, in turn, became the source of the snail for Burma, Singapore, Thailand [11], and possibly Vietnam [1], and all three countries have the emergent haplotype C. Singapore became the source of the snail in Borneo [39], and all individuals sampled there also carried the C haplotype. A similar case to India also took place in Hawaii where the snails descended from two individuals introduced there from Taiwan (Formosa) in 1936 [40], which in turn received the snail from Singapore after 1917 [11]. The Philippines also most likely obtained *A. fulica* from Taiwan via Japanese soldiers during the Second World War in 1942 [41]. As the *A. fulica* populations in Hawaii, Singapore and the Philippines harbor the C haplotype, the snails in Taiwan would therefore also be expected to harbor this emergent haplotype. In French Polynesia, *A. fulica* was first recorded in Tahiti in 1967 and later in other areas in 1978. These snails supposedly came from Asia [1], though it is not clear from which country. Nevertheless, the presence of the emergent haplotype C in Moorea and Tahiti mirrors the lack of genetic variation in the majority of introduced populations in Asia and other parts of the world.

*Achatina fulica* came to Brazil as a result of recent introduction to raise snails for food [7], [16], [17]. Initial populations of *A. fulica* in the country were thought to have originated from Indonesia [7], but it is suspected that the Brazilian *A. fulica* actually came from multiple sources (D. Robinson pers. comm.). This possibility could explain the presence of haplotypes C and D, both of which are also found in Mayotte and Mauritius and raises the question as to the actual sources of these South American populations. The possible presence of other haplotypes in South America should also not be discounted.

North America, particularly Florida, has been experiencing an outbreak of *A. fulica* since 2011, and the USDA is currently involved in massive efforts to eradicate the invader. The population sampled from Florida, all of which bear haplotype C, came from a core group of reproducing individuals suspected by agricultural authorities to have originated from the West Indies, where the same haplotype is also found (D. Robinson pers. comm.).

The median-joining network demonstrated that the non-African/Indian Ocean haplotypes E (Philippines), F (New Caledonia and Barbados), P (India) and Q (Ecuador) were derived from haplotype C by one substitutional mutation for each. These haplotypes are most likely recent mutations that arose from the emergent C haplotype outside of East Africa and the Indian Ocean islands. However, we cannot discount the possibility that they represent previously undetected haplotypes from East Africa or the Indian Ocean islands and that we failed to detect them due to limited sampling, particularly in East Africa.

The relationship of the East African haplotypes from Tanzania and Uganda with those from the Indian Ocean islands is less clear. It is believed that the Giant African Land Snail was introduced first in Madagascar from East Africa prior to 1800, and it is these Madagascan *A. fulica* snails that were eventually introduced to nearby islands [1], [8] such as Mayotte. This raises some very important questions. Is haplotype C found in mainland East Africa? If so, then this would suggest that C was originally present in East Africa and was brought first to Madagascar, which harbours C, and to nearby islands such as Mayotte. If C is *not* present in mainland East Africa, then this haplotype could have originated on islands off East Africa, possibly on Madagascar. This question also applies to the other Mayotte haplotypes. Are they also found in Madagascar and mainland East Africa? All these questions necessitate further sampling in East Africa and in Madagascar, which the current study could not achieve due to severe governmental restrictions to sample in these areas as well as the limited number of collectors who could go in the field. However, Emberton et al. [42] noted that *A. fulica* is widespread in Tanzania and could be found in montane and lowland forest habitats. The presence of six haplotypes in Dar Es Salaam alone, particularly in disturbed habitats, points to the existence of more haplotypes of the snail in East Africa, with the strong possibility that the source population of C in the continent has not yet been identified.

The lack of genetic variation in populations outside of East Africa and the Indian Ocean islands clearly indicate bottlenecking on the basis of the 16S rRNA gene. Most of the snails from these populations were derived from the emergent haplotype C from the Indian Ocean islands and have been spreading for more than 200 years. Outside of South America where both C and D were found, there is no evidence to suggest that multiple introductions involving different haplotypes from the native range have taken place.

In conclusion, 18 haplotypes were identified from populations of *A. fulica*, with two emergent haplotypes coming from the Indian Ocean islands: predominantly haplotype C across the globe and haplotype D in South America. Four non-African/Indian Ocean haplotypes (E from the Philippines, F from New Caledonia and Barbados, P from India and Q from Ecuador) were derived from haplotype C as shown by median-joining network analysis. Populations outside East Africa and the Indian Ocean islands generally exhibited a lack of genetic variation based on the 16S rRNA marker, implying the possibility of bottlenecking in these populations. Further sampling in the native range is therefore required to check for the presence of the source populations of the emergent haplotypes as well as the non-African/Indian Ocean island haplotypes.

## Supporting Information

File S1 **Kits used for the methodology.**
(DOCX)
