# Supplementary material for: Restricted Genetic Variation in Populations of Achatina (Lissachatina) fulica outside of East Africa and the Indian Ocean Islands Points to the Indian Ocean Islands as the Earliest Known Common Source
Source: PLoS One. 2014 Sep 9;9(9):e105151. doi: 10.1371/journal.pone.0105151 (PMC4159197; doi:10.1371/journal.pone.0105151)
Supplement: File S1 — Kits used for the methodology. (DOCX) [file pone.0105151.s001.docx]

Supporting Information

The PCR amplification was carried out using BIOTAQ^TM^ Red DNA polymerase or ROCHE^TM^ Taq DNA polymerase. SSCP analysis was carried out using a native polyacrylamide gel system (7.5 ml 2X MDE^TM^ gel solution of Cambrex Bio Science®, USA.
